# Kinetics and Muscle Activity Patterns during Unweighting and Reloading Transition Phases in Running

**DOI:** 10.1371/journal.pone.0168545

**Published:** 2016-12-19

**Authors:** Patrick Sainton, Caroline Nicol, Jan Cabri, Joëlle Barthèlemy-Montfort, Pascale Chavet

**Affiliations:** 1 Aix-Marseille University, CNRS, ISM UMR 7287, Marseille, France; 2 Department of Physical Performance, Norwegian School of Sport Sciences, Oslo, Norway; 3 LU:NEX-University, Faculty Health Sciences, Differdange, Luxembourg; West Virginia University, UNITED STATES

## Abstract

Amongst reduced gravity simulators, the lower body positive pressure (LBPP) treadmill is emerging as an innovative tool for both rehabilitation and fundamental research purposes as it allows running while experiencing reduced vertical ground reaction forces. The appropriate use of such a treadmill requires an improved understanding of the associated neuromechanical changes. This study concentrates on the runner’s adjustments to LBPP-induced unweighting and reloading during running. Nine healthy males performed two running series of nine minutes at natural speed. Each series comprised three sequences of three minutes at: 100% bodyweight (BW), 60 or 80% BW, and 100% BW. The progressive unweighting and reloading transitions lasted 10 to 15 s. The LBPP-induced unweighting level, vertical ground reaction force and center of mass accelerations were analyzed together with surface electromyographic activity from 6 major lower limb muscles. The analyses of stride-to-stride adjustments during each transition established highly linear relationships between the LBPP-induced progressive changes of BW and most mechanical parameters. However, the impact peak force and the loading rate systematically presented an initial 10% increase with unweighting which could result from a passive mechanism of leg retraction. Another major insight lies in the distinct neural adjustments found amongst the recorded lower-limb muscles during the pre- and post-contact phases. The preactivation phase was characterized by an overall EMG stability, the braking phase by decreased quadriceps and soleus muscle activities, and the push-off phase by decreased activities of the shank muscles. These neural changes were mirrored during reloading. These neural adjustments can be attributed in part to the lack of visual cues on the foot touchdown. These findings highlight both the rapidity and the complexity of the neuromechanical changes associated with LBPP-induced unweighting and reloading during running. This in turn emphasizes the need for further investigation of the evolution over time of these neuromechanical changes.

## Introduction

As gravity plays an essential role in terrestrial locomotion, several studies since the early works on weightlessness [1–3] have investigated the effects of reduced gravity on locomotion [4], balance control [5] and proprioceptive information [6]. The recent development of reduced gravity simulators [4] provides the opportunity to study the neuromechanical adjustments to partial unweighting over repeated running cycles. One major difference with microgravity testing conditions is that only the supporting limbs experiences a simulated reduction of gravity.

Amongst reduced gravity simulators, the lower body positive pressure (LBPP) treadmill is emerging as an innovating tool allowing safe running in the early stages of post-injury recovery with a quicker restoration of the gait pattern [7]. The LBPP treadmill creates an adjustable lifting force via an airtight chamber applied distally to the person’s pelvis leading to a partial reduction of the subject’s bodyweight (BW). In agreement with previous unweighting studies using a harness device [8], the LBPP-induced unweighting is also known to result in increased flight and stride durations with a limited decrease in contact time, and also in decreased vertical ground reaction forces [9]. However, although the active peak force is reported to decrease almost linearly with the bodyweight reduction, the impact peak force and the loading rate may not systematically decrease, especially above 75% bodyweight [9]. Considering the reported association of such factors with running injuries [10], additional information is thus needed to clarify the underlying neuro-mechanical mechanisms.

The muscle activation changes associated with unweighting reported in the literature are rather limited and divergent. Based on a global electromyography (EMG) analysis per stride, Liebenberg et al. [11] reported a preserved activation pattern due to the observation of similar EMG decreases for the recorded thigh and shank muscles. However, the distinction of the stance and flight phases revealed inter-muscular differences in the unweighting-induced EMG decrease, with no significant change in the hamstring activation during the stance phase [12]. Similarly Jensen et al. [13] reported unchanged hamstring activation and larger decreases in the vasti than in the triceps surae muscle activation. The additional distinction of the preactivation, braking and push-off phases revealed an unchanged preactivation of the triceps surae muscle group followed by a large decrease, but during the push-off phase only [14]. This latter study also revealed after-effects when returning to normal bodyweight suggesting rapid updates of the internal model of the running pattern. Moreover similar triceps surae neural adjustments and kinetic changes were found after 30 s and 3 min of running in the unweighting as well as in the reloading conditions. This early adoption of a different running pattern highlighted the need for a more detailed time course examination of the runners’ adjustments to the LBPP treadmill condition.

For both fundamental and clinical purposes, the LBPP treadmill is of particular interest as the transition phases (between full BW and either 80 or 60% BW) last 10–15 s allowing progressive adjustments to occur during their time course. There is still a surprising lack of literature about the time course of the associated neuromechanical changes. When considering adjustments to short-term perturbations, most studies examined the kinetic and kinematic adjustments to sudden rather than to progressive ground condition changes when running at normal BW on an uneven surface [15,16]. In particular, center of mass, leg length and leg stiffness as well as leg angle of attack (at touch down) were found to be adjusted within one to three steps only. In accordance to the conservative spring-mass model [17–19], the angle of attack is considered as being passively and proportionally adjusted to the flight duration, through leg retraction in the late swing phase [20]. On the other hand, preactivation control is still considered as a key for altering leg posture depending on flight duration [21] and on ground level [16]. These findings thus put into question the reported unchanged triceps surae preactivation while facing large increases in both flight height and flight duration in unweighted running conditions [14]. Passive adjustments of the leg properties with the unweighting-induced flight increase may be considered sufficient to cope with the BW changes. However, the absence of changes in the triceps surae preactivation may be attributed to the runners’ lack of experience in unweighted running [14]. Furthermore, neural adjustments may have occurred only temporarily and in other (unrecorded) muscle groups.

Therefore, the present study was focused on the neuromechanical adjustments to partial unweighting and reloading transition phases while running on a LBPP treadmill. We hypothesized that the progressive nature of the LBPP-induced unweighting and reloading would favor the occurrence of stride-to-stride mechanical adjustments of the temporal, kinetic and EMG running patterns. Our second hypothesis was that transient changes in preactivation would occur, especially during the somewhat uncommon unweighting transition phase. Our third hypothesis was that distinct neural adjustments would take place amongst lower limb muscle groups.

## Materials and Methods

### Subjects

Nine recreational male runners volunteered for this study (mean age 21.3 ± 3.7 years, mean height 172.6 ± 6.1 cm, mean body mass 65.4 ± 7.0 kg). The inclusion criteria required that all volunteers were free from any previous or present lower limb and back injuries. All volunteers were bilateral rearfoot-strike runners. To avoid the reported differences in muscle activation between rear- and forefoot strikers [22], the striking pattern of all volunteers was checked on this specific treadmill at 100BW one to two days prior to the testing protocol. This session was also used to individually determine each subject’s preferred speed. Ethical approval for this study was obtained from the ethics committee of Aix-Marseille University. According to the Declaration of Helsinki, all procedures were carried out with the adequate understanding and written consent of the volunteer participants.

### Experimental design

**Fig 1:**
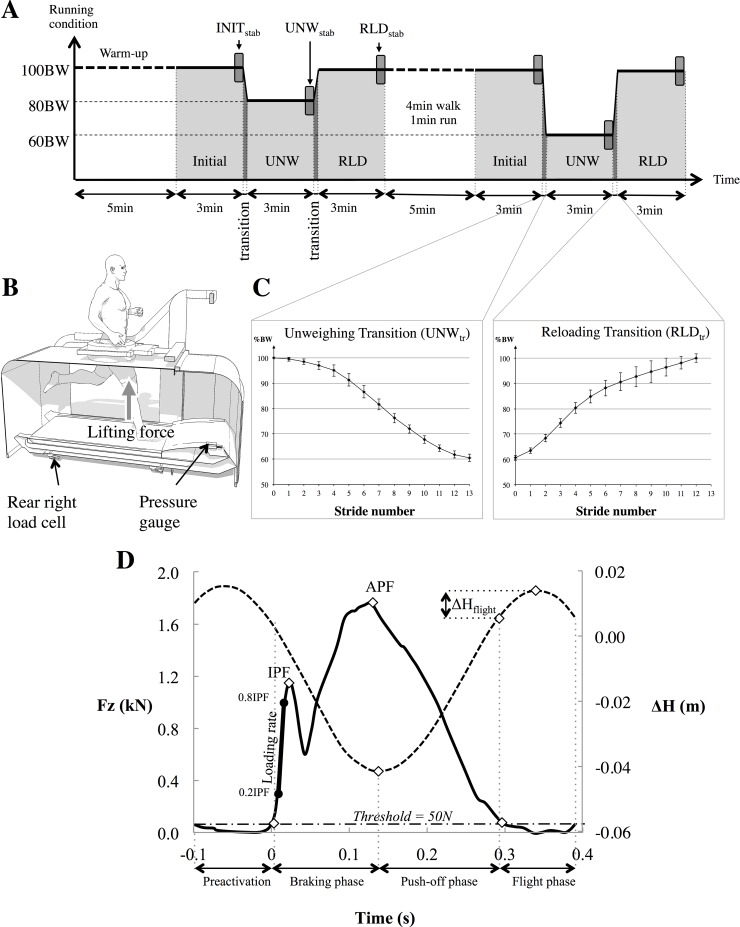
Testing protocol, Experimental set-up and typical trace at 100BW. Testing protocol (A) performed on the LBPP treadmill (B) which allows running at either normal bodyweight (100BW) or in unweighting conditions. The protocol included two randomized running series performed at 80 and 60% bodyweight (80BW and 60BW, respectively). Each series included three conditions, referred as INITIAL, unweighted (UNW) and reloaded (RLD). The unweighting and reloading transitions (UNW_tr_ and RLD_tr_, respectively) were induced by a progressive increase and decrease in pressure of the LBPP chamber (C). Frame D shows an individual example of the vertical ground reaction force (Fz) (solid line) and vertical center of mass displacement (ΔH) (dashed line) during the preactivation, contact phase (composed of braking and push-off phases) and flight phase. IPF initial peak force, APF active peak force, and ΔH_flight_ vertical center of mass displacement during the flight.

The participants ran at their preferred speed (2.45 ± 0.16 m.s^-1^) on an instrumented treadmill (M310 Anti-gravity Treadmill^®^, AlterG Inc., Fremont, CA.), which allowed them to run either at 100% bodyweight (100BW) or in unweighting conditions at 80% (80BW) and 60% bodyweight (60BW) (Fig 1A). The specific running speed for each individual subject remained unchanged during each running series. The AlterG^®^ treadmill device applies a substantial lifting force via an LBPP device comprising an airtight chamber fixed distally to the subject’s iliac crest (Fig 1B). The LBPP system induces a small increase in air pressure around the user’s lower body to create a lifting force at the level of the waist, so that the lower limbs still experience Earth’s gravity [23]. All volunteers wore flexible neoprene shorts with a waist seal zipped to the chamber and the same flat-soled shoes with no orthotics. Before the testing protocol, a calibration was performed to adjust the chamber pressure while the subject was standing on the treadmill. A 15 min familiarization run was performed at 100BW one to two days prior to the testing protocol.

The testing protocol (Fig 1A) started with a 5 min warm-up period to reach the individual preferred running speed at 100BW and was followed by two running series of 9 min. Each running series included 3 successive conditions of 3 min, with the initial and reloaded (RLD) conditions performed at 100BW, and the intermediate unweighting condition (UNW at either 80BW or 60BW in a randomized order). The unweighting and reloading transition phases (UNW_tr_ and RLD_tr_, respectively) in between running conditions were initiated progressively and lasted for 10.1 ± 0.7 s and 14.9 ± 1.1 s in the 80BW and 60BW running series, respectively (Fig 1C). The runners were informed of the imminent transition prior to the UNW_tr_ and RLD_tr_ initiation. The two running series were separated by a 4 min walk and a 1 min run at preferred speed (Fig 1A).

### Measurements

The air pressure variation inside the LBPP chamber was measured using a pressure gauge (MPXV5010DP, Freescale^®^, Inc., Austin, TX.) to assess indirectly the partial body support provided by the AlterG^®^ technology. The differential pressure (P_atmospheric_-P_chamber_) and the instantaneous treadmill velocity were recorded simultaneously with the vertical ground reaction force (Fz) obtained from 4 dynamic bonded foil strain gauge load cells. These are designed to accurately measure compression loads and are rated to 4500 N (XA–shear beam load cell, Sentran^®^, Ontario, CA) and located under the frame of the AlterG^®^ treadmill. The AlterG® treadmill was previously stiffened to shift its natural vibration frequency to close to 40 Hz. These data were sampled at 1 kHz using an A/D board (NI_USB 6212 BNC, National Instrument^®^, Inc., Austin, TX.). A tri-axial accelerometer (± 6 g, Trigno, Delsys^®^, Inc., Natick, MA.; sampling frequency: 148.1 Hz) was taped at the level of the 1^st^ sacral vertebrae and was used to record indirectly the 3D accelerations of the center of mass.

Surface electromyographic signals (EMG) of the soleus (SOL), gastrocnemius medialis and lateralis (GaM, GaL) and tibialis anterior (TA) as well as the vasti medialis (VM) and lateralis (VL) muscles of the left lower limb were recorded at 2 kHz (Trigno, Delsys^®^, Inc., Natick, MA.) with a Common Mode Rejection Rate > 80 dB, 20–450 Hz ± 10% bandwidth and 1000V/V gain. Positioning of the active surface electrodes was carried out according to SENIAM recommendations [24]. A rising edge trigger was used to synchronize the EMG and accelerometer data recorded by the Delsys^®^ software (EMGwork^®^, Delsys^®^, Inc., Natick, MA.) with the vertical force, speed and pressure signals issued from the AlterG® treadmill.

All signals were recorded using a Virtual Instrument developed in Labview (v.8.5, National Instruments^®^, Inc., Austin, TX).

### Data analysis

To examine the time course of the neuromechanical adjustments, the left lower limb temporal, kinetic, kinematic and EMG data analyses were carried out during 30 s at the end of each running condition to characterize the stabilized running patterns (INIT_stab_, UNW_stab_ and RLD_stab_ on Fig 1A) as well as for each of the successive left strides during the transitions (UNW_tr_ and RLD_tr_ on Fig 1A and 1C). Each of these selected 30 s periods included on average 42 ± 2 successive left strides at 100BW, 38 ± 2 at 80BW and 36 ± 3 at 60BW. Due to the different time durations of the transition phases in the 80BW and 60BW running series, they included on average 8 ± 1 and 13 ± 1 left strides, respectively.

The vertical ground reaction force analysis included impact peak force (IPF), loading rate from 0.2 to 0.8 IPF and active peak force (APF) during the contact phase (Fig 1D). The beginning and the end of the contact phase were determined from the vertical ground reaction force signal using a threshold set at 50 N. Braking and push-off phases were identified from the minimum of the double integral (displacement) of the vertical center of mass acceleration, using a 1–12 Hz two-way band-pass 2^nd^ order Butterworth damped filter. These data enabled us to calculate the braking and push-off phase durations, their corresponding mean force values and the APF. The vertical center of masse excursion was calculated during the flight (ΔH_flight_). Contact duration (T_contact_) and flight duration (T_flight_) of the left lower limb were used to calculate its stride duration (T_stride_) (Fig 1D).

The obtained EMG signals were band-pass filtered (20–400 Hz), rectified and low-pass filtered using a 75 Hz critically damped filter [25]. Both integrated and averaged muscle activities were then calculated for the total contact period as well as for the preactivation, braking and push-off phases (i.e. the 3 stretch-shortening cycle phases). The preactivation phase was defined as the 100 ms preceding ground contact [26].

### Statistical analysis

The two running series were analyzed separately as well as the unweighting and the reloading phases. The analyses focused on the left stride data. All measured variables were normally distributed (Shapiro-Wilk test) (Statistica®, 12.0, StatSoft®, Inc., Tulsa, Oklahoma).

The effect of the unweighting was analyzed separately for the stable and transition states. Two-tailed paired *t* tests were used to compare the mean values of the last 30 s of the initial running condition (INIT_stab_), first, to the values of each stride during the unweighting transition (UNW_tr_), and second, to the last 30 s values of the unweighted condition (UNW_stab_) (Fig 1A). For clarification reasons, the group averaged transition values present the last stride values only. Similarly, the last stride values of the transition were compared to the corresponding mean values once stabilized (UNW_stab_).

For the analyses of the reloading effect, the mean values of the last 30 s of the unweighting condition were used as a reference (UNW_stab_ in Fig 1A) and again the last stride values were compared to the corresponding mean values once stabilized (RLD_stab_). Statistical analyses and graphics were performed in a similar way for the UNW_tr_.

In order to further characterize the relationship between the changes in bodyweight and stride parameters, Pearson product-moment correlation coefficients were calculated to measure the degree of linear dependence during the UNW_tr_ and RLD_tr_.

When a statistical difference was found between two conditions, the meaningfulness of the effect size was assessed with Cohen’s d (small effect size: 0.2 ≤ d < 0.5, medium effect size: 0.5 ≤ d < 0.8, large effect size: 0.8 ≤ d) [27].

## Results

Following the approach of Sainton et al. [14], mechanical data are expressed as median and interquartile range. For clarification reason the EMG changes are expressed as mean (± standard error). All data are presented with statistical p and Cohen’s d values. The original dataset (S1 Dataset) can be requested by contacting the corresponding author.

### Unweighting- and reloading-induced mechanical changes

**Fig 2:**
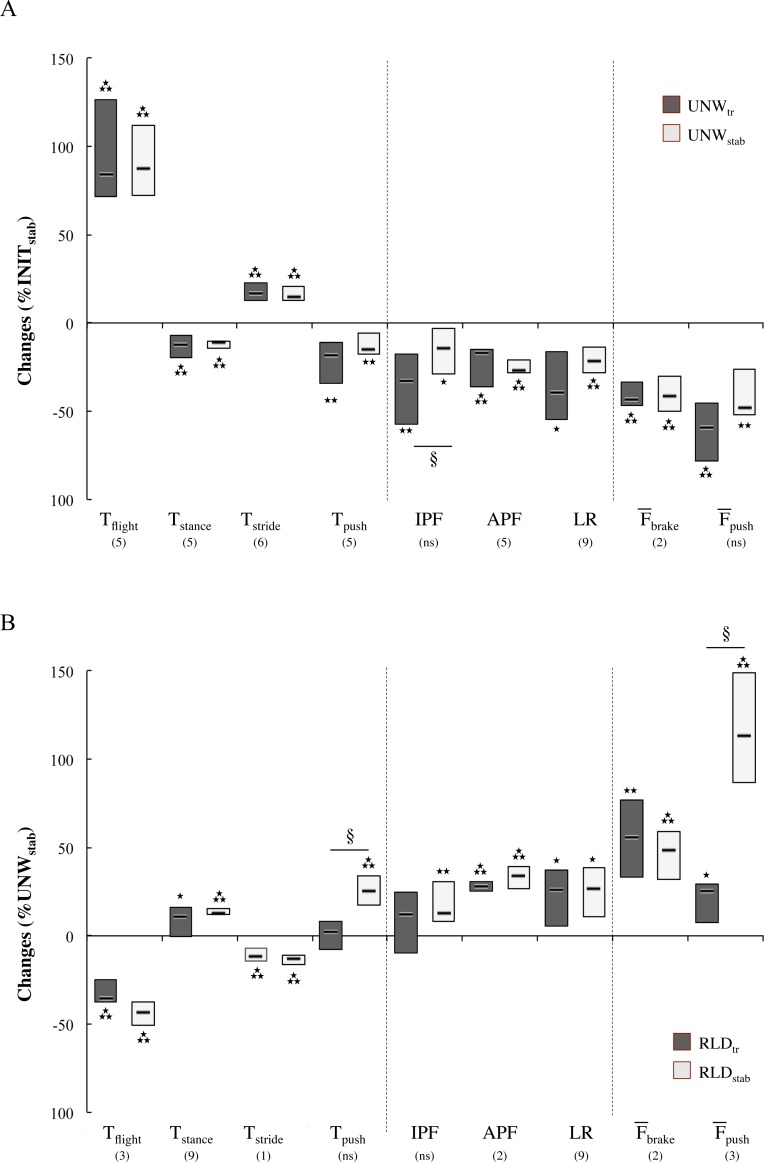
(A): Unweighting-induced changes (Δ% INIT_stab_) in the temporal, kinetic and kinematic stride characteristics at the end of the transition phase (UNW_tr_) and once stabilized (UNW_stab_) at 60BW. (B): Reloading-induced changes (Δ% UNW_stab_) in the same parameters. For each variable, the median and interquartile range represents the individual changes. *p < 0.05 and **p < 0.01 when statistically different from their reference values (INIT_stab_ and UNW_stab_, respectively). The stride number corresponding to the onset of significant change is indicated in between parentheses. Significant differences between the last stride of the transition and the mean values once stabilized are indicated by § with p < 0.05.

As illustrated in Fig 2A for the 60BW series, most of the significant unweighting-induced changes in the temporal, kinetic and kinematic running parameters measured once stabilized (UNW_stab_) were already accomplished by the end of the unweighting transition (UNW_tr_). The 80BW series presented similar findings (S1 Fig) except for the IPF and loading rate parameters which did not vary significantly during the UNW_tr_, but showed subsequent reductions once the running pattern stabilized (IPF: - 12% (- 24.3 to– 8.2), p < 0.01, d = 0.3 and Loading rate: -15% (-28.2 to -10.8), p < 0.01, d = 0.3). Reloading (Fig 2B) was associated with significant and opposite changes to those induced by unweighting, except push-off time (T_push_) and impact peak force (IPF), which did not vary during the RLD_tr_, increasing only thereafter. Similarly to what was observed during unweighting, braking time (T_brake_) did not vary significantly.

**Fig 3:**
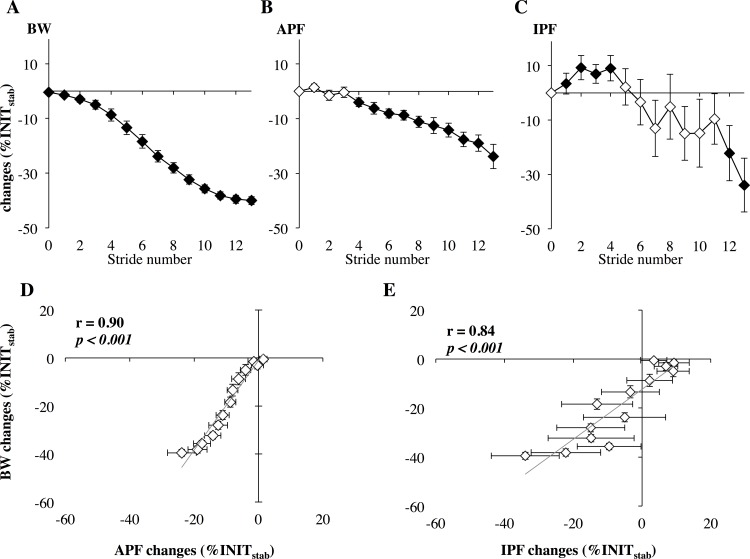
The upper panel shows changes in (A) bodyweight (BW), (B) active peak force (APF) and (C) impact peak force (IPF) along the 13 strides of the UNWtr in the 60BW running series. Group mean (+/- standard error) differences from the initial condition values at 100BW (INIT_stab_) are presented as closed points (in black) when significant at p < 0.05. The lower panels (D and E) show the correlations between changes in BW and those of either APF (D) or IPF (E) in the 60BW running series.

The stride-to-stride analysis within the transition phase revealed that most parameters such as APF decreased progressively (Fig 3B) with the smooth reduction of bodyweight (Fig 3A). As illustrated for the APF parameter (Fig 3D), significant and positive relationships were found between the decrease of bodyweight and most of the mechanical stride parameters, whereas negative relationships were found for T_flight_, T_stride_ and ΔH_flight_ (Table 1). The impact peak force (IPF) and the loading rate presented a particular behavior, with a significant increase of 9% (-2 to 13) up to the 5th stride for the IPF (p < 0.039, d = 1.3) and of 10% (4 to 16) up to the 4th stride for the loading rate (p < 0.040, d = 5.5) before subsequently decreasing progressively (Fig 3C for the IPF). As illustrated on Fig 3 for the IPF, these parameters also presented a large inter-individual variability and a low correlation coefficient with the bodyweight decrease compared with other parameters such as the APF (Fig 3E *vs* 3D).

**Table 1:** Pearson product-moment correlation coefficients (r) between LBPP-induced relative changes of bodyweight and of stride mechanical parameters.

			T_stride_	T_flight_	ΔH_flight_	T_stance_	T_push_	IPF	APF	F_brake_	F_push_
80BW	UNW_tr_	r	-0.84	-0.86	-0.71	0.82	0.86	0.59	0.82	0.70	0.86
		p	**	**	**	**	***	ns	*	**	***
	RLD_tr_	r	-0.77	-0.86	-0.68	0.66	0.44	0.64	0.87	0.81	0.69
		p	***	***	**	**	**	*	***	***	**
60BW	UNW_tr_	r	-0.91	-0.91	-0.86	0.77	0.68	0.84	0.9	0.89	0.73
		p	***	***	***	***	***	***	***	***	***
	RLD_tr_	r	-0.89	-0.86	-0.84	0.69	0.41	0.5	0.91	0.88	0.69
		p	***	***	***	***	*	**	***	***	***

The significance level (p) of the correlations is presented by * with p < 0.05, ** with p < 0.01 and *** with p < 0.001.

### Unweighting-induced neural changes

**Fig 4:**
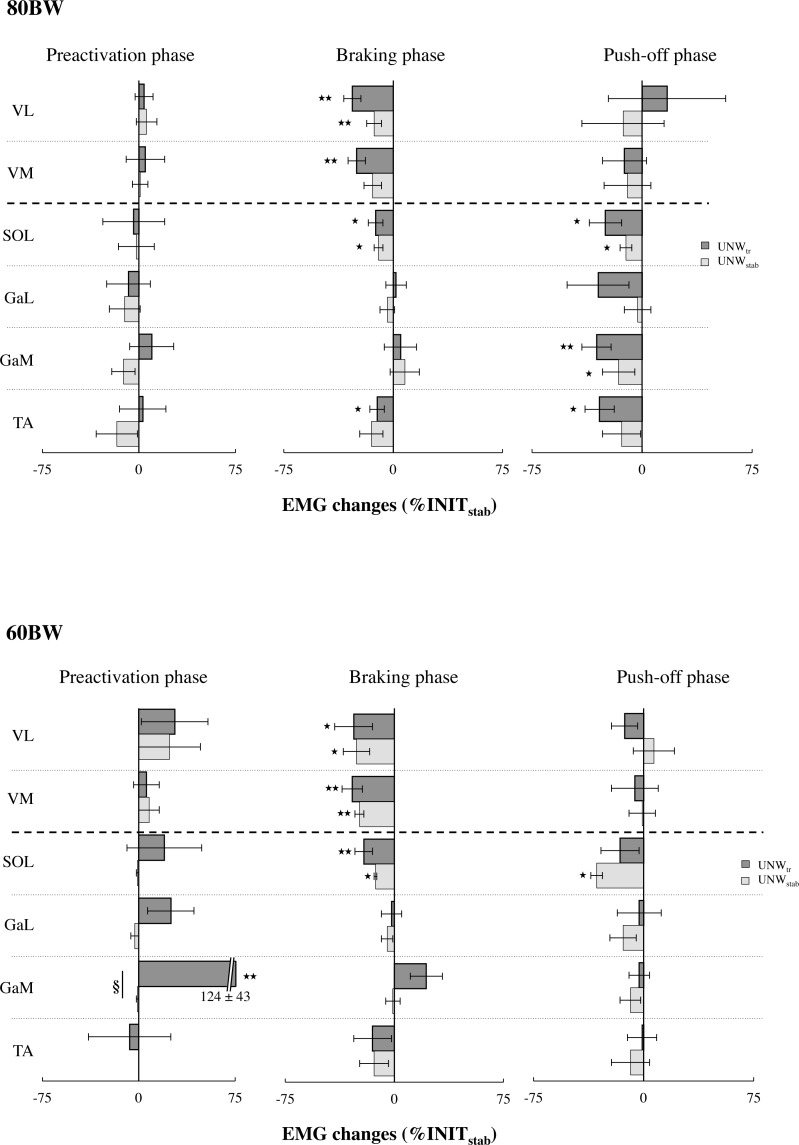
Unweighting-induced changes (% INIT_stab_) in the EMG activity of the thigh and shank muscles at the end of the transition phase (UNW_tr_) and once stabilized (UNW_stab_) at 80BW (upper graph) and at 60BW (lower graph). Significant group mean (**±** standard error) differences are presented with * p < 0.05 and ** p < 0.01 when statistically different from the reference INIT_stab_ values. GAM value is indicated numerically as it goes well beyond the chosen scale. Significant differences between the last stride of the transition and the mean values once stabilized are indicated by § with p < 0.05.

The results of the EMG data analysis (Table 2) are shown in Fig 4 for unweighting-induced changes in the EMG activity of the thigh and shank muscles at the end of the transition phase (UNW_tr_) and once stabilized (UNW_stab_) in the 80BW and 60BW conditions, respectively. No significant change in EMG activity was found during the preactivation phase, except for the GaM muscle that showed a large increase of preactivation after the 8^th^ stride of the UNW_tr_ in the 60BW series (124 ± 43%, p < 0.010, d = 4.1). This change of GaM preactivation was negatively correlated to the decrease in bodyweight during the UNW_tr_ (r = -0.79; p < 0.001). No significant change in muscle preactivation was observed, once the stabilized condition was reached.

**Table 2:** Unweighting-induced changes (% INIT_stab_) in mean EMG activity of the recorded left limb muscles during the transition (UNW_tr_) and once stabilized (UNW_stab_) in the 80BW and 60BW series.

			TA	GaM	GaL	SOL	VM	VL
Preactivation	80BW	UNW_tr_	3 ± 18	10 ± 17	-8 ± 17	-4 ± 24	5 ± 15	4 ± 7
			ns	ns	ns	ns	ns	ns
		UNW_stab_	-17 ± 16	-12 ± 9	-11 ± 12	-2 ± 14	1 ± 6	6 ± 8
			ns	ns	ns	ns	ns	ns
	60BW	UNW_tr_	-7 ± 32	**124 ± 43**	25 ± 18	20 ± 29	6 ± 10	28 ± 26
			ns	***0*.*010***^***l***^ **(8)**	ns	ns	ns	ns
		UNW_stab_	0 ± 10	-1 ± 6	-3 ± 7	-1 ± 8	8 ± 11	24 ± 11
			ns	ns	ns	ns	ns	ns
Braking phase	80BW	UNW_tr_	**-11 ± 5**	5 ± 11	2 ± 7	**-12 ± 5**	**-25 ± 6**	**-28 ± +6**
			***0*.*036***^***m***^ **(6)**	ns	ns	***0*.*018***^***l***^ **(5)**	***0*.*003***^***l***^ **(4)**	***0*.*002***^***l***^ **(3)**
		UNW_stab_	-15 ± 8	8 ± 10	-4 ± 5	**-10 ± 3**	-14 ± 6	-13 ± 5
			ns	ns	ns	***0*.*029***^***s***^	ns	ns
	60BW	UNW_tr_	-15 ± 13	22 ± 11	-2 ± 7	**-21 ± 6**	**-29 ± 7**	**-28 ± 13**
			ns	ns	ns	***0*.*004***^***l***^ **(8)**	***0*.*003***^***l***^ **(8)**	***0*.*033***^***l***^ **(8)**
		UNW_stab_	-14 ± 10	-1 ± 5	-5 ± 4	-13 ± 1	-24 ± 3	-26 ± 9
			ns	ns	ns	*0*.*016*^*s*^	ns	ns
Push-off phase	80BW	UNW_tr_	**-29 ± 10**	**-31 ± 10**	-30 ± 21	**-25 ± 11**	-12 ± 15	17 ± 40
			***0*.*011***^***l***^ **(3)**	***0*.*006***^***l***^ **(6)**	ns	***0*.*012***^***l***^ **(6)**	ns	ns
		UNW_stab_	-14 ± 13	**-16 ± 11**	-3 ± 9	-11 ± 4	-10 ± 16	-13 ± 18
			ns	***0*.*034***^***s***^	ns	ns	ns	ns
	60BW	UNW_tr_	-1 ± 10	-3 ± 7	-3 ± 15	-16 ± 13	-6 ± 16	-13 ± 9
			ns	ns	ns	ns	ns	ns
		UNW_stab_	-9 ± 13	-9 ± 7	-14 ± 9	**-32 ± 4**	-1 ± 9	7 ± 14
			ns	ns	ns	***0*.*034***^***s***^	ns	ns

Group mean (± standard error) differences from INIT_stab_ are presented with their statistical p values in italic. The non-significant changes are denoted as ns. The stride number corresponding to the onset of two subsequent significant changes is indicated within brackets. Cohen’s d level is indicated as the superscript “s” for small, “m” for medium or “l” for large.

**Fig 5:**
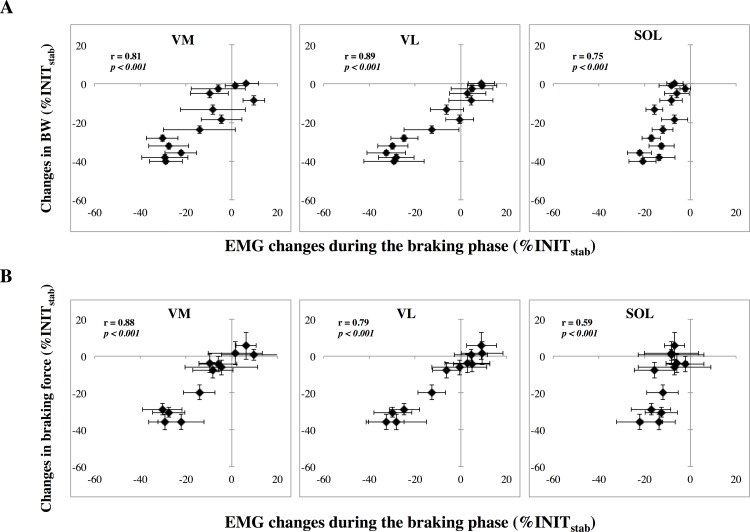
Significant relationships between the changes in VM, VL and SOL mean muscle activities in the braking phase and (A) the bodyweight changes or (B) the mean braking force changes during the UNW_tr_ in the 60BW running series. r: Pearson coefficient, p: statistical level.

The braking phase analysis revealed progressive EMG reductions during the UNW_tr_ for the VL, VM and SOL muscles in both running series as well as for the TA muscle in the 80BW series (Fig 4). In both running series the onset of the significant change occurred between the 3^rd^ and the 8^th^ strides (Table 2). High positive correlations (0.75 < r < 0.89; p < 0.001) were found between the changes in SOL and vasti muscle activation with the changes in bodyweight (Fig 5A). Both VM and VL changes in activation were also positively related to the mean braking force changes (Fig 5B). Once stabilized (UNW_stab_), VL and SOL muscle activities remained significantly decreased in both running series. The VM muscle activity was decreased only in the 60BW series.

The push-off phase analysis revealed limited neural changes in the two running series (Fig 4). The 80BW series presented during the UNW_tr_ significant EMG decreases (p < 0.05, 2.3 < d < 4.1) after the 3^rd^ stride for TA and after the 6^th^ stride for SOL and GaM muscles. The 60BW series presented larger interindividual variability with an initially reduced SOL muscle activity from the 5^th^ to the 8^th^ stride (p < 0.05, d = 2.9). Only SOL muscle activity remained reduced in both running series once stabilized.

### Reloading-induced neural changes

The EMG data analysis (Table 3) did not reveal any significant change during the preactivation phase, the transition or when once stabilized.

**Table 3:** Reloading-induced changes (% UNW_stab_) in mean EMG activity of the recorded left limb muscles during the transition (RLD_tr_) and once stabilized (RLD_stab_) in the 80BW and 60BW series.

			TA	GaM	GaL	SOL	VM	VL
Preactivation	80BW	RLD_tr_	41 ± 32	-21 ± 10	15 ± 37	4 ± 17	3 ± 8	7 ± 22
			ns	ns	ns	ns	ns	ns
		RLD_stab_	31 ± 24	-10 ± 12	6 ± 10	13 ± 11	5 ± 1	1 ± 14
			ns	ns	ns	ns	ns	ns
	60BW	RLD_tr_	10 ± 23	2 ± 28	3 ± 15	11 ± 30	5 ± 19	-11 ± 11
			ns	ns	ns	ns	ns	ns
		RLD_stab_	28 ± 42	1 ± 14	4 ± 15	7 ± 18	4 ±5	12 ± 18
			ns	ns	ns	ns	ns	ns
Braking phase	80BW	RLD_tr_	44 ± 37	-4 ± 5	-6±7	**9 ± 3**	20 +18	**36 ± 13**
			ns	ns	ns	***0*.*011***^***l***^ **(6)**	ns	***0*.*013***^***l***^ **(3)**
		RLD_stab_	16 ± 10	0 ± 7	4 ± 4	**13 ± 2**	7 ± 8	**29 ± 8**
			ns	ns	ns	***0*.*007***^***l***^	ns	***0*.*001***^***l***^
	60BW	RLD_tr_	21 ± 24	5 ± 9	6 ± 4	**18 ± 5**	**59 ± 18**	22 ± 12
			ns	ns	ns	***0*.*003***^***l***^ **(5)**	***0*.*007***^***l***^ **(8)**	ns
		RLD_stab_	14 ±14	9 ± 7	6 ± 4	**22 ± 3**	47 ± 7	29 ± 22
			ns	ns	ns	***0*.*035***^***l***^	ns	ns
Push-off phase	80BW	RLD_tr_	15 ± 38	0 ± 8	10 ± 21	34 ± 20	8 ± 17	12 ± 13
			ns	ns	ns	ns	ns	ns
		RLD_stab_	17 ± 11	**21 ± 8**	**7 ± 4**	25 ± 6	11 ± 13	13 ± 23
			ns	***0*.*02***^***l***^	***0*.*019***^***m***^	ns	ns	ns
	60BW	RLD_tr_	2 ± 19	4 ± 12	5 ± 14	20 ± 17	12 ± 29	4 ± 12
			ns	ns	ns	ns	ns	ns
		RLD_stab_	16 ± 18	8 ± 7	12 ± 10	50 ± 17	25 ± 8	13 ± 23
			ns	ns	ns	ns	ns	ns

Group mean (± standard error) differences from UNW_stab_ are presented with their statistical p values in italic. The non-significant changes are denoted as ns. The stride number corresponding to the onset of two subsequent significant changes is indicated within brackets. Cohen’s d level is indicated as the superscript “s” for small, “m” for medium or “l” for large.

The braking phase analysis for the RLD_tr_ revealed increased muscle activities in SOL and VL in the 80BW series and in SOL and VM in the 60BW series. As previously observed in the UNW_tr_ (Fig 5, upper graph), changes in SOL and vasti muscles activities were positively related to BW changes (SOL: r = 0.80, VM: r = 0.88 and VL: r = 0.79; p < 0.001). Once stabilized, SOL muscle activity remained increased in both running series whereas VL muscle activity was increased only in the 80BW series (Table 3).

Regarding the push-off phase, no significant EMG changes occurred during the RLDtr. In the 80BW series, the gastrocnemii muscle activities remained significantly increased once stabilized (Table 3).

## Discussion

This study aimed to assess the neuromechanical adjustments to partial unweighting and reloading while running on LBPP treadmills, with special emphasis on the transition phases. In support of our first hypothesis, all mechanical changes of the running pattern except the IPF were found to vary linearly with the LBPP-induced changes in BW, so that the running pattern adjustment was mostly completed during the UNW_tr_ and RLD_tr_ phases. Our second hypothesis of transient changes in preactivation, especially during the UNW_tr_, is only supported by the large increase in GaM preactivation in the 60BW running series. Confirming our third hypothesis, distinct neural adjustments were found to take place in the quadriceps and shank muscle activities during the braking and push-off phases.

In agreement with earlier LBPP studies, the analysis of the unweighting running pattern once stabilized confirmed the large increase in flight time and the slight decrease in contact time leading to a fall in stride frequency [23,28]. Unweighting resulted also in nearly proportional decrease of the active peak (APF) vertical force [8,9,23]. The present transition phase analyses demonstrate for the first time the linearity of the relationships between the LBPP-induced progressive changes of BW with most of the mechanical stride parameters (Fig 5A) and reveal mirrored changes during the UNW_tr_ and RLD_tr_ phases. In both transitions phases, BW changes were highly and positively related to those of APF, mean braking and push-off forces as well as push-off time and contact time whereas strong negative relationships were found with the flight height, flight time and stride time changes. Providing support to passive adjustments of the leg properties in accordance with the conservative spring-mass model [17–19], the EMG analysis revealed for most muscles no change in the centrally programmed preactivation during the transition phases. During the UNW_tr_, the LBPP-induced progressive elevation of the runner’s center of mass during the flight phase is suggested to have resulted in a steeper angle of attack at ground impact. This so-called leg retraction is reported to result in nearly constant leg stiffness without any change in muscle activity [20]. Mirrored passive mechanisms are expected to have occurred during the progressive RLD_tr_. For the rehabilitation domain, it should be mentioned that the lack of significant change of the impact peak force (IPF) during the UNW_tr_ phase resulted from its initial and systematic increase up to the 5th stride before its progressive decrease thereafter (Fig 3C). In this vein, similar observations of reduced APF but unchanged IPF have been reported once the unweighting running pattern was stabilized [23,28], but data were lacking for the transition phase. The present EMG analysis did not reveal any significant change in preactivation that could explain the initial 10% increase in IPF. To further investigate the initial IPF increase, additional investigation is still needed to identify the lower limb segment position during transition. This however should not be a problem for most clinical protocols which start and stop in the unweighting condition. Except for this parameter, the highly linear relationships found between the LBPP-induced progressive changes of BW with most mechanical stride parameters demonstrate the stride-to-stride adjustments to unweighting and reloading.

Our second hypothesis on transient changes in preactivation especially during the UNW_tr_ is only supported by the delayed, but steadily increased GaM muscle preactivation in the 60BW running series. This preactivation increase occurred too late to explain the initial rise in IPF. As previously mentioned, the unchanged preactivation of most muscles reinforces the hypothesis of the major role of passive mechanisms during the pre-impact phase along both transition phases [20]. However, this lack of neural adjustment may also be considered as reflecting opposite influences on the preactivation. Decreased preactivation has been reported in the case of unweighting [29–31]. On the other hand, the LBPP-induced increases in flight height and duration could have resulted in an increased preactivation to tolerate the expected larger impact load as previously reported in running and jumping [26,32,33]. Another influence lies in the lack of visual cues on the actual foot touchdown on LBPP type treadmill, which may explain the observed selective increase of GaM preactivation. Such an increase has indeed been reported by Müller et al. [21] in case of increased flight time in the absence of visual cues. In the present 60BW series, this GaM increase occurred with no associated change of TA preactivation. The absence of decrease in TA activity suggests the adoption of a midfoot rather than a heel striking running pattern [22]. This expected shift of the striking pattern may also have contributed to the significantly delayed decrease of IPF. Three minutes after the UNW_tr_ phase (UNW_stab_), IPF was found to be partially re-increased while GaM preactivation had dropped down, back to its level when running at normal BW. Despite the absence of visual cues, the RLD_tr_ phase of the 60BW running series did not lead to any specific change in preactivation. This reinforces the overall stability of preactivation during the LBPP-induced unweighting and reloading transitions and confirms also our previous findings in the stabilized unweighting running condition [14]. For the clinical domain, this preactivation stability should be checked when repeating practice sessions.

Concerning to our third hypothesis, our results revealed rather specific activation changes of the thigh (vasti) and shank (TA and triceps surae) muscles. During the UNW_tr_, progressive EMG decreases were observed mostly in the vasti muscles during the braking phase and in the shank muscles during the push-off phase (in the 80BW series only for the SOL muscle). Similar patterns were observed once the unweighting running pattern was stabilized confirming the triceps surae neural adjustments previously reported [14]. However, these findings differ distinctly from the overall decrease in EMG activity with no change in the activation pattern as reported by Liebenberg et al. [11]. This discrepancy may be attributed to the quantification of the global muscle activity per stride in the aforementioned study, rather than per stretch-shortening cycle phase (preactivation, braking and push-off) used in our studies. Our analysis of the braking phase revealed contrasting EMG changes: Unchanged triceps surae activation as compared to large vasti activation decreases. Most importantly, vasti activation changes were highly and positively related to the LBPP-induced unweighting and to the resulting mean braking force changes. These findings demonstrate that the decrease in braking force during the UNW_tr_ did not result from the LBPP-induced unweighting only, but also from a progressive reduction in the vasti muscle activation. In both UNW_tr_ and RLD_tr_, soleus muscle activation was found to vary in a similar manner as the vasti, but differently from the gastrocnemii. These findings are in line with the reported monosynaptic coupling between muscles operating at the knee and ankle joints, in particular between the soleus and the vasti muscles rather than between the soleus and the gastrocnemii muscles [34,35]. Specific activation of knee extensors and soleus muscle, but depressed activity of the biarticular gastrocnemii muscle have been reported in case of simultaneous plantar flexion and knee extension [36]. The push-off phase analysis revealed during the UNW_tr_ phase a selective decrease of the triceps surae activation in the 80BW running series only. In agreement with our previous study [14], this was also observed once the running pattern was stabilized. Considering the potential use of LBPP to enhance recovery in patients following lower limb surgery [37], the specificity of the EMG patterns should be taken into consideration for the appropriate use of the LBPP treadmill running protocols in the rehabilitation programs.

A few limitations in our methods need to be addressed. First, the limited number of participants and the low level of the self-selected running speeds. However, all measured variables presented a normal distribution and quite low inter individual differences in the mechanical changes with unweighting and reloading. To improve the understanding of the neuromuscular adjustments and of the initial IPF increase, 3D force-measurements should be combined with lower limb kinematics. However, due to the inflated chamber, such a combination remains a methodological challenge on the AlterG® treadmill.

We have previously established with healthy runners that acute kinematic, kinetic and EMG after-effects appear once returning to normal bodyweight running [14]. This suggests acute updates of the internal model of the running pattern. The present EMG analyses revealed stretch-shortening cycle phase-dependent adjustments as well as muscle group-dependent neural adjustments to short-term unweighting and reloading. These observations require reexamination along repeated LBPP treadmill sessions such as those used in rehabilitation. In addition, high frequency ultrasonography while running [38] could be used to quantify the acute muscle-tendon responses in order to complete our understanding of the functional benefits and limits of the unweighing exercising protocols used in rehabilitation.

## Conclusion

In conclusion, this LBPP treadmill running study highlights the mirrored neuromechanical adjustments to unweighting and reloading. The specific analysis of the successive strides of the transition phases demonstrates for the first time the linearity of the relationships between the LBPP-induced bodyweight changes with most of the mechanical stride parameters. Furthermore, the EMG analyses revealed stretch-shortening cycle phase-dependent adjustments as well as muscle group-dependent neural adjustments to short-term unweighting and reloading. These neural adjustments can be partly attributed to the lack of visual cues on the actual foot touchdown. Considering the use of the LBPP treadmill in rehabilitation, these muscle group and phase specific EMG patterns should be taken into consideration and their evolution over time carefully investigated.

## Supporting Information

S1 Dataset Excel table of dataset. Dataset including all the individual mean data per testing running session and for each stabilized and transition running periods of interest.
(XLS)

S1 Fig Unweighting- and reloading-induced changes in the temporal, kinetic and kinematic stride characteristics at the end of the transition phase and once stabilized at 80BW. (A): Unweighting-induced changes (Δ% INIT_stab_) in the temporal, kinetic and kinematic stride characteristics at the end of the transition phase (UNW_tr_) and once stabilized (UNW_stab_) at 80BW. (B): Reloading-induced changes (Δ% UNW_stab_) in the same parameters. For each variable, the median and interquartile range represents the individual changes. *p < 0.05 and **p < 0.01 when statistically different from their reference values (INIT_stab_ and UNW_stab_, respectively). The stride number corresponding to the onset of significant change is indicated in between parentheses. Significant differences between the last stride of the transition and the mean values once stabilized are indicated by § with p < 0.05.
(TIF)

## Abbreviations

100BW: 100% bodyweight
60BW: 60% bodyweight
80BW: 80% bodyweight
APF: Active peak force
BW: Bodyweight
ΔH_flight_: Vertical displacement of center of mass during the flight phase
EMG: Electromyography
$\bar{F}$_brake_: Mean vertical force during the braking phase
$\bar{F}$_push_: Mean vertical force during the push-off phase
GaL: Gastrocnemius lateralis
GaM: Gastrocnemius medialis
INIT_stab_: Stabilized phase of the initial running condition
IPF: Impact peak force
LBPP: Lower body positive pressure
RLD: Reloading condition
RDL_stab_: Reloading stabilized phase
RDL_tr_: Reloading transition phase
SOL: Soleus
TA: Tibialis anterior
T_contact_: Contact duration
T_flight_: Flight duration
UNW: Unweighting condition
UNW_stab_: Unweighting stabilized phase
UNW_tr_: Unweighting transition phase
VL: Vastus lateralis
VM: Vastus medialis

## References

[1] Massion J, Fabre JC, Mouchnino L, Obadia A. Body orientation and regulation of the center of gravity during movement under water. J Vestib Res Equilib Orientat. 1995;5: 211–221. 7627380

[2] Roll R, Gilhodes JC, Roll JP, Popov K, Charade O, Gurfinkel V. Proprioceptive information processing in weightlessness. Exp Brain Res. 1998;122: 393–402. 9827858 10.1007/s002210050527

[3] Newman DJ, Alexander HL, Webbon BW. Energetics and mechanics for partial gravity locomotion. Aviat Space Environ Med. 1994;65: 815–823. 7818450

[4] Sylos-Labini F, Lacquaniti F, Ivanenko YP. Human Locomotion under Reduced Gravity Conditions: Biomechanical and Neurophysiological Considerations,. BioMed Res Int BioMed Res Int. 2014;2014, 2014: e547242. 10.1155/2014/547242 PMC4163425 25247179

[5] Ritzmann R, Freyler K, Weltin E, Krause A, Gollhofer A. Load Dependency of Postural Control-Kinematic and Neuromuscular Changes in Response to over and under Load Conditions. PloS One. 2015;10: e0128400 10.1371/journal.pone.0128400 26053055 PMC4459704

[6] Lowrey CR, Perry SD, Strzalkowski NDJ, Williams DR, Wood SJ, Bent LR. Selective skin sensitivity changes and sensory reweighting following short-duration space flight. J Appl Physiol. 2014;116: 683–692. 10.1152/japplphysiol.01200.2013 24458748

[7] Tenforde AS, Watanabe LM, Moreno TJ, Fredericson M. Use of an antigravity treadmill for rehabilitation of a pelvic stress injury. PM R. 2012;4: 629–631. 10.1016/j.pmrj.2012.02.003 22920318

[8] Chang YH, Huang HW, Hamerski CM, Kram R. The independent effects of gravity and inertia on running mechanics. J Exp Biol. 2000;203: 229–238. 10607533 10.1242/jeb.203.2.229

[9] Grabowski AM, Kram R. Effects of velocity and weight support on ground reaction forces and metabolic power during running. J Appl Biomech. 2008;24: 288–297. 18843159 10.1123/jab.24.3.288

[10] Zadpoor AA, Nikooyan AA. The relationship between lower-extremity stress fractures and the ground reaction force: A systematic review. Clin Biomech. 2011;26: 23–28. 10.1016/j.clinbiomech.2010.08.005 20846765

[11] Liebenberg J, Scharf J, Forrest D, Dufek JS, Masumoto K, Mercer JA. Determination of muscle activity during running at reduced body weight. J Sports Sci. 2011;29: 207–214. 10.1080/02640414.2010.534806 21170806

[12] Hunter I, Seeley MK, Hopkins JT, Carr C, Franson JJ. EMG activity during positive-pressure treadmill running. J Electromyogr Kinesiol. 2014;24: 348–352. 10.1016/j.jelekin.2014.01.009 24613660

[13] Jensen BR, Hovgaard-Hansen L, Cappelen KL. Muscle Activation and Estimated Relative Joint Force During Running With Weight Support on a Lower-Body Positive Pressure Treadmill. J Appl Biomech. 2016; 32(4):335–41. 10.1123/jab.2015-0075 26957520

[14] Sainton P, Nicol C, Cabri J, Barthelemy-Montfort J, Berton E, Chavet P. Influence of short-term unweighing and reloading on running kinetics and muscle activity. Eur J Appl Physiol. 2015;115: 1135–1145. 10.1007/s00421-014-3095-3 25566954

[15] Ferris DP, Louie M, Farley CT. Running in the real world: adjusting leg stiffness for different surfaces. Proc R Soc Lond B Biol Sci. 1998;265: 989–994. 10.1098/rspb.1998.0388 PMC1689165 9675909

[16] Müller R, Grimmer S, Blickhan R. Running on uneven ground: leg adjustments by muscle pre-activation control. Hum Mov Sci. 2010;29: 299–310. 10.1016/j.humov.2010.01.003 20304516

[17] Blickhan R, Seyfarth A, Geyer H, Grimmer S, Wagner H, Günther M. Intelligence by mechanics. Philos Transact A Math Phys Eng Sci. 2007;365: 199–220. 10.1098/rsta.2006.1911 17148057

[18] Biewener AA, Daley MA. Unsteady locomotion: integrating muscle function with whole body dynamics and neuromuscular control. J Exp Biol. 2007;210: 2949–2960. 10.1242/jeb.005801 17704070 PMC2651961

[19] Grimmer S, Ernst M, Günther M, Blickhan R. Running on uneven ground: leg adjustment to vertical steps and self-stability. J Exp Biol. 2008;211: 2989–3000. 10.1242/jeb.014357 18775936

[20] Müller R, Blickhan R. Running on uneven ground: leg adjustments to altered ground level. Hum Mov Sci. 2010;29: 578–589. 10.1016/j.humov.2010.04.007 20591519

[21] Müller R, Häufle DFB, Blickhan R. Preparing the leg for ground contact in running: the contribution of feed-forward and visual feedback. J Exp Biol. 2015;218: 451–457. 10.1242/jeb.113688 25524978

[22] Landreneau LL, Watts K, Heitzman JE, Childers WL. Lower limb muscle activity during forefoot and rearfoot strike running techniques. Int J Sports Phys Ther. 2014;9: 888–897. 25540704 PMC4275193

[23] Donelan JM, Kram R. Exploring dynamic similarity in human running using simulated reduced gravity. J Exp Biol. 2000;203: 2405–2415. 10903155 10.1242/jeb.203.16.2405

[24] Hermens HJ, Freriks B, Disselhorst-Klug C, Rau G. Development of recommendations for SEMG sensors and sensor placement procedures. J Electromyogr Kinesiol Off J Int Soc Electrophysiol Kinesiol. 2000;10: 361–374. 10.1016/s1050-6411(00)00027-4 11018445

[25] Morio C, Nicol C, Barla C, Barthèlemy J, Berton E. Acute and 2 days delayed effects of exhaustive stretch-shortening cycle exercise on barefoot walking and running patterns. Eur J Appl Physiol. 2012;112: 2817–2827. 10.1007/s00421-011-2242-3 22124522

[26] Komi PV, Gollhofer A, Schmidtbleicher D, Frick U. Interaction between man and shoe in running: considerations for a more comprehensive measurement approach. Int J Sports Med. 1987;8: 196–202. 10.1055/s-2008-1025655 3623781

[27] Cohen J. Statistical power analysis for the behavioral sciences Hillsdale, N.J.: L. Erlbaum Associates; 1988.

[28] He JP, Kram R, McMahon TA. Mechanics of running under simulated low gravity. J Appl Physiol Bethesda Md 1985. 1991;71: 863–870. 10.1152/jappl.1991.71.3.863 1757322

[29] Gollhofer A, Kyröläinen H. Neuromuscular control of the human leg extensor muscles in jump exercises under various stretch-load conditions. Int J Sports Med. 1991;12: 34–40. 10.1055/s-2007-1024652 2030057

[30] Avela J, Santos PM, Kyröläinen H, Komi PV. Effects of different simulated gravity conditions on neuromuscular control in drop jump exercises. Aviat Space Environ Med. 1994;65: 301–308. 8002909

[31] Avela J, Komi PV, Santos PM. Effects of differently induced stretch loads on neuromuscular control in drop jump exercise. Eur J Appl Physiol. 1996;72: 553–562. 10.1007/BF00242290 8925831

[32] Schmidtbleicher D, Gollhofer A. Neuromuskuläre Untersuchungen zur Bestimmung individueller Belastungsgrößen für ein Tiefsprungtraining. Leist-Sport. 1982;12: 298–307.

[33] Santello M, McDonagh MJ. The control of timing and amplitude of EMG activity in landing movements in humans. Exp Physiol. 1998;83: 857–874. 9782194 10.1113/expphysiol.1998.sp004165

[34] Meunier S, Pierrot-Deseilligny E, Simonetta M. Pattern of monosynaptic heteronymous Ia connections in the human lower limb. Exp Brain Res. 1993;96: 534–544. 8299754 10.1007/BF00234121

[35] Lamy J-C, Iglesias C, Lackmy A, Nielsen JB, Katz R, Marchand-Pauvert V. Modulation of recurrent inhibition from knee extensors to ankle motoneurones during human walking. J Physiol. 2008;586: 5931–5946. 10.1113/jphysiol.2008.160630 18936080 PMC2655432

[36] Suzuki T, Chino K, Fukashiro S. Gastrocnemius and soleus are selectively activated when adding knee extensor activity to plantar flexion. Hum Mov Sci. 2014;36: 35–45. 10.1016/j.humov.2014.04.009 24922619

[37] Patil S, Steklov N, Bugbee WD, Goldberg T, Colwell CW, D’Lima DD. Anti-gravity treadmills are effective in reducing knee forces. J Orthop Res. 2013;31: 672–679. 10.1002/jor.22272 23239580

[38] Sano K, Nicol C, Akiyama M, Kunimasa Y, Oda T, Ito A, et al Can measures of muscle-tendon interaction improve our understanding of the superiority of Kenyan endurance runners? Eur J Appl Physiol. 2015;115: 849–859. 10.1007/s00421-014-3067-7 25476746

