# Correction: Kinetics and Muscle Activity Patterns during Unweighting and Reloading Transition Phases in Running

**DOI:** 10.1371/journal.pone.0176508

**Published:** 2017-04-20

**Authors:** Patrick Sainton, Caroline Nicol, Jan Cabri, Joëlle Barthèlemy-Montfort, Pascale Chavet

There are errors in Table 2. Please see the corrected Table 2 here.

**Table 2:** Unweighting-induced changes (% INIT_stab_) in mean EMG activity of the recorded left limb muscles during the transition (UNW_tr_) and once stabilized (UNW_stab_) in the 80BW and 60BW series.

			TA	GaM	GaL	SOL	VM	VL
Preactivation	80BW	UNW_tr_	3 ± 18	10 ± 17	-8 ± 17	-4 ± 24	5 ± 15	4 ± 7
			ns	ns	ns	ns	ns	ns
		UNW_stab_	-17 ± 16	-12 ± 9	-11 ± 12	-2 ± 14	1 ± 6	6 ± 8
			ns	ns	ns	ns	ns	ns
	60BW	UNW_tr_	-7 ± 32	**124 ± 43**	25 ± 18	20 ± 29	6 ± 10	28 ± 26
			ns	***0*.*010***^***l***^ **(8)**	ns	ns	ns	ns
		UNW_stab_	0 ± 10	-1 ± 6	-3 ± 7	-1 ± 8	8 ± 11	24 ± 11
			ns	ns	ns	ns	ns	ns
Braking phase	80BW	UNW_tr_	**-11 ± 5**	5 ± 11	2 ± 7	**-12 ± 5**	**-25 ± 6**	**-28 ± +6**
			***0*.*036***^***m***^ **(6)**	ns	ns	***0*.*018***^***l***^ **(5)**	***0*.*003***^***l***^ **(4)**	***0*.*002***^***l***^ **(3)**
		UNW_stab_	-15 ± 8	8 ± 10	-4 ± 5	**-10 ± 3**	-14 ± 6	**-13 ± 5**
			ns	ns	ns	***0*.*029***^***s***^	ns	***0*.*049***^***s***^
	60BW	UNW_tr_	-15 ± 13	22 ± 11	-2 ± 7	**-21 ± 6**	**-29 ± 7**	**-28 ± 13**
			ns	ns	ns	***0*.*004***^***l***^ **(8)**	***0*.*003***^***l***^ **(8)**	***0*.*033***^***l***^ **(8)**
		UNW_stab_	-14 ± 10	-1 ± 5	-5 ± 4	**-13 ± 1**	**-24 ± 2**	**-26 ± 9**
			ns	ns	ns	***0*.*016***^***s***^	***0*.*018***^***s***^	***0*.*039***^***l***^
Push-off phase	80BW	UNW_tr_	**-29 ± 10**	**-31 ± 10**	-30 ± 21	**-25 ± 11**	-12 ± 15	17 ± 40
			***0*.*011***^***l***^ **(3)**	***0*.*006***^***l***^ **(6)**	ns	***0*.*012***^***l***^ **(6)**	ns	ns
		UNW_stab_	-14 ± 13	**-16 ± 11**	-3 ± 9	**-11 ± 4**	-10 ± 16	-13 ± 18
			ns	***0*.*034***^***s***^	ns	***0*.*035***^***s***^	ns	ns
	60BW	UNW_tr_	-1 ± 10	-3 ± 7	-3 ± 15	-16 ± 13	-6 ± 16	-13 ± 9
			ns	ns	ns	ns	ns	ns
		UNW_stab_	-9 ± 13	-9 ± 7	-14 ± 9	**-32 ± 4**	-1 ± 9	7 ± 14
			ns	ns	ns	***0*.*034***^***s***^	ns	ns

Group mean (± standard error) differences from INIT_stab_ are presented with their statistical p values in italic. The non-significant changes are denoted as ns. The stride number corresponding to the onset of two subsequent significant changes is indicated within brackets. Cohen’s d level is indicated as the superscript “s” for small, “m” for medium or “l” for large.
