# Supplementary figures and images for: Kinetics and Muscle Activity Patterns during Unweighting and Reloading Transition Phases in Running
Source: PLoS One. 2016 Dec 19;11(12):e0168545. doi: 10.1371/journal.pone.0168545 (PMC5167401; doi:10.1371/journal.pone.0168545)

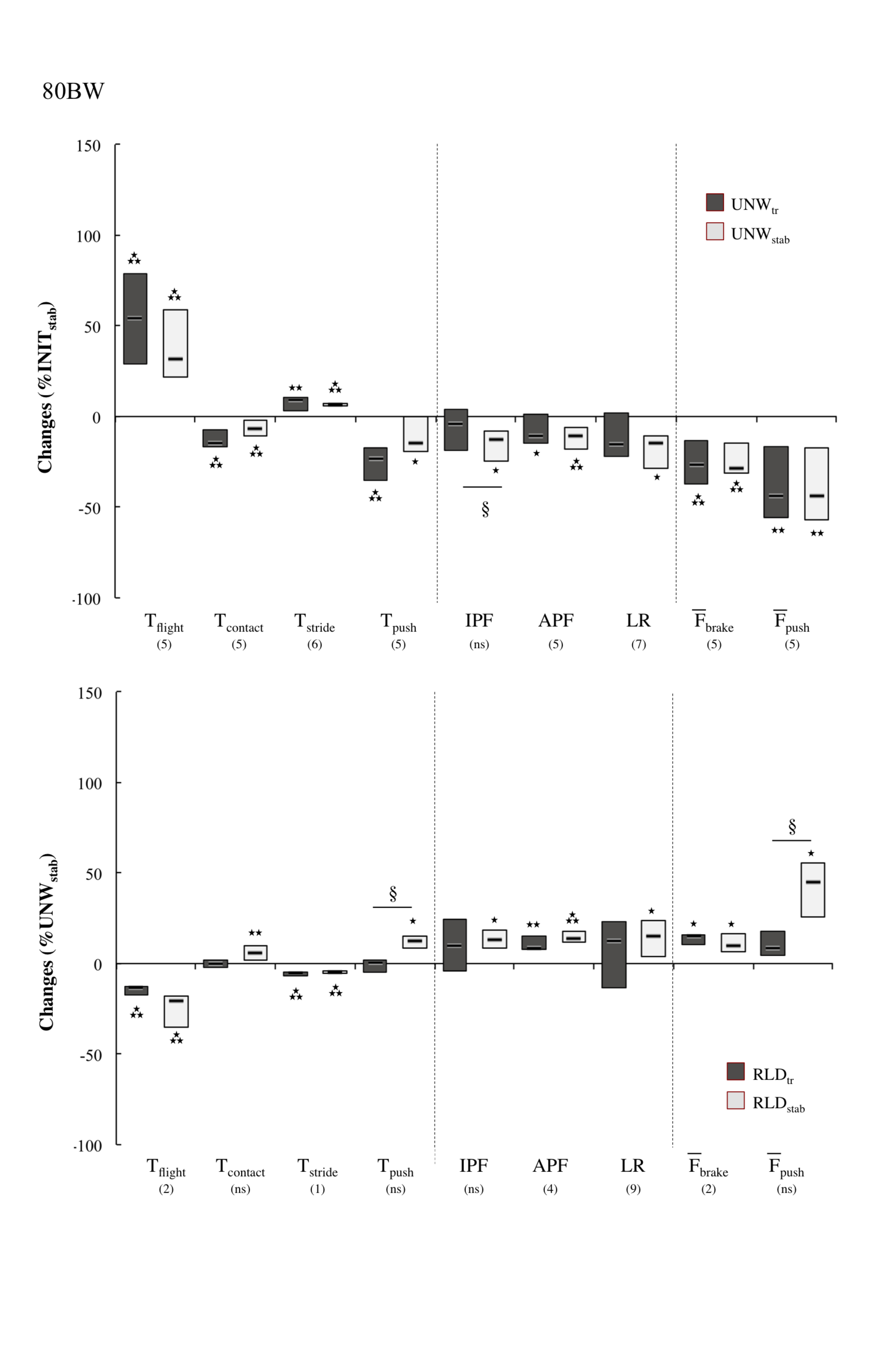

Supplement: S1 Fig — (A): Unweighting-induced changes (Δ% INITstab) in the temporal, kinetic and kinematic stride characteristics at the end of the transition phase (UNWtr) and once stabilized (UNWstab) at 80BW. (B): Reloading-induced changes (Δ% UNWstab) in the same parameters. For each variable, the median and interquartile range represents the individual changes. *p < 0.05 and **p < 0.01 when statistically different from their reference values (INITstab and UNWstab, respectively). The stride number corresponding to the onset of significant change is indicated in between parentheses. Significant differences between the last stride of the transition and the mean values once stabilized are indicated by § with p < 0.05. (TIF) [file pone.0168545.s002.tif]
